# Community-based scheduled screening and treatment of malaria in pregnancy for improved maternal and infant health in The Gambia, Burkina Faso and Benin: study protocol for a randomized controlled trial

**DOI:** 10.1186/1745-6215-15-340

**Published:** 2014-08-28

**Authors:** Susana Scott, Petra F Mens, Halidou Tinto, Alain Nahum, Esmée Ruizendaal, Franco Pagnoni, Koen Peeters Grietens, Lindsay Kendall, Kalifa Bojang, Henk Schallig, Umberto D’Alessandro

**Affiliations:** Disease Control and Elimination Theme, Medical Research Council Unit, Fajara, The Gambia; Infectious Disease Epidemiology Department, London School of Hygiene & Tropical Medicine, London, UK; Royal Tropical Institute/Koninklijk Instituut voor de Tropen, Parasitology Unit, Amsterdam, The Netherlands; Clinical Research Unit of Nanoro, URCN/CMA, Centre Muraz, Ouagadougou, Burkina Faso; Centre de Recherches Entomologiques de Cotonou, Cotonou, Benin; Special Programme for Research and Training in Tropical Diseases (TDR), World Health Organization, Geneva, Switzerland; Institute of Tropical Medicine, Antwerp, Belgium; Nagasaki University School of International Health Development, Nagasaki, Japan

**Keywords:** Malaria, Pregnancy, Community based treatment, Rapid diagnostic tests, Sulfadoxine-pyrimethamine, Sub-Saharan Africa, Artemether-lumefantrine

## Abstract

**Background:**

In sub-Saharan Africa, malaria continues to cause over 10,000 maternal deaths and 75,000 to 200,000 infant deaths. Successful control of malaria in pregnancy could save lives of mothers and babies and is an essential part of antenatal care in endemic areas. The primary objective is to determine the protective efficacy of community-scheduled screening and treatment (CSST) using community health workers (CHW) against the primary outcome of prevalence of placental malaria. The secondary objectives are to determine the protective efficacy of CSST on maternal anaemia, maternal peripheral infection, low birth weight, selection of sulfadoxine-pyrimethamine (SP) resistance markers, and on antenatal clinic (ANC) attendance and coverage of intermittent preventive treatment during pregnancy (IPTp-SP).

**Methods/design:**

This is a multi-centre cluster-randomised controlled trial involving three countries with varying malaria endemicity; low (The Gambia) versus high transmission (Burkina Faso and Benin), and varying degrees of SP resistance (high in Benin and moderate in Gambia and Burkina Faso). CHW and their related catchment population who are randomised into the intervention arm will receive specific training on community-based case management of malaria in pregnancy. All women in both study arms will be enrolled at their first ANC visits in their second trimester where they will receive their first dose of IPTp-SP. Thereafter, CHW in the intervention arm will perform scheduled monthly screening and treatment in the womens homes. At time of delivery, a placental biopsy will be collected from all women to determine placental malaria. At each contact point, filter paper and blood slides will be collected for detection of malaria infection and SP resistance markers.

**Discussion:**

To reach successful global malaria control, there is an urgent need to access those at greatest risk of malaria infection. The project is designed to develop a low-cost intervention in pregnant women which will have an immediate impact on the malaria burden in resource-limited countries. This will be done by adding to the standard IPTp-SP delivered through the health facilities: an “extension” strategy to the communities in rural areas thus bringing health services closer to where women live.

**Trial registration:**

Current Controlled Trials: ISRCTN37259296 (5 July 2013), and clinicaltrials.gov: NCT01941264 (10 September 2013).

## Background

Malaria infection remains a major public health problem in (sub) tropical regions throughout the world. Approximately half of the world’s population is at risk of malaria and every year this leads to about 207 million clinical malaria cases [1].

In endemic countries, pregnant women have a higher risk of malaria compared to other adults due to the physiological hormonal changes and reduced immunity. In areas of stable, moderate to intense transmission, *Plasmodium falciparum* infection in pregnancy is often asymptomatic, yet it is an important contributor to maternal morbidity, preterm delivery, perinatal morbidity and mortality [2, 3] and may be associated with an increased risk of post-partum haemorrhage [4]. In areas of low, often unstable, transmission, malaria infection can evolve towards severe disease and result in maternal and foetal death. Such adverse outcomes are more frequent in primigravidae [5], in HIV-infected women [6] or in those from non-endemic countries as they do not have any immunity acquired before pregnancy [7]. The increased susceptibility of pregnant women to infection, and most specifically of primigravidae, is explained by the sequestration of *P. falciparum* in the placenta due to its affinity with the chondroitin sulphate A receptor. Placental malaria causes intrauterine growth retardation and the delivery of low birth weight babies who have a significantly higher risk of dying before their first birthday due to the reduced transfer of essential nutrients to the developing foetus as a result of the placental infection [7].

In sub-Saharan Africa, over 30 million women living in malaria endemic areas become pregnant each year [8], with malaria causing an estimated >10,000 maternal deaths and 75,000 to 200,000 infant deaths. In addition, it is responsible for 2 to 15% of maternal anaemia cases and up to 14% of low birth weight babies resulting in poor growth and development [9]. Furthermore, increasing evidence shows that placental malaria *per se* is associated with an increased risk of malaria infection and anaemia in the first and second year of life of the infant [10, 11], possibly mediated by the exposure to blood-stage malaria antigens *in utero* and the acquisition of a tolerant phenotype that persists into childhood [12]. Importantly, these effects are independent of low birth weight; that is, they can occur in any child born to mothers with placental malaria, and seem to be more pronounced in multigravidae [10]. If these observations are confirmed, the burden of malaria in African pregnant women would extend beyond that observed in primigravidae and could be vastly underestimated.

Successful control of malaria in pregnancy could save lives of mothers and babies and is an essential part of antenatal care in endemic areas [2, 3]. For sub-Saharan Africa, the World Health Organization has developed guidelines for the control of malaria in pregnancy consisting of a three-pronged approach:

1. Prompt and effective management of uncomplicated malaria in the second and third trimester with artemisinin-based combination therapy. For the first trimester, it is recommended to combine quinine with clindamycin [13].
2. Long-lasting insecticidal bed nets for all pregnant women in malaria-endemic countries [14].
3. Intermittent preventive treatment during pregnancy with sulfadoxine-pyrimethamine (IPTp-SP) at regular intervals during pregnancy [15]. This is a simple, pragmatic policy that has proven very valuable in areas with stable, moderate to high malaria transmission. SP is safe when given in the second and third trimester of pregnancy, widely available, cheap and well tolerated. IPTp-SP can clear any existing malaria infection and provides post-treatment prophylaxis for a certain period of time, depending on SP sensitivity of local parasites [2].

Unfortunately, the coverage of long-lasting insecticidal bed nets and IPTp-SP in many sub-Saharan African countries remains low [14, 16], particularly in peripheral areas with difficult access to health facilities, leaving certain remote, poor populations out of reach of basic services. To add further complexities, SP resistance is increasing and, thus, there is the urgent need to develop alternatives or additional efforts to the current IPTp-SP policy [17].

### Study objectives

The project is designed to develop a low-cost intervention for pregnant women that will have an immediate impact on the malaria burden in resource-limited countries. In this study, it is proposed to add an “extension” strategy to the communities in rural areas in addition to the standard IPTp-SP delivered through health facilities. The novelty of this study is to bring health services closer to where women live, using community health workers (CHWs) to provide an antimalarial intervention to women with difficult access to the formal health system. The study will build on the use of CHWs who are already managing malaria cases within the framework of community case management of malaria (CCMm) [18].

The main aim is to establish whether maternal and infant health can be improved by adding to existing IPTp-SP at antenatal clinics (ANCs) community-scheduled screening and treatment (CSST) for malaria among pregnant women through CHWs. The study will test the primary hypothesis that CSST plus IPTp-SP will reduce placental malaria compared to IPTp-SP alone.

- Primary objective: To determine the protective efficacy of CSST using CHWs against the primary outcome of prevalence of placental malaria.
- Secondary objectives: To determine the protective efficacy of CSST on maternal anaemia, maternal peripheral infection, low birth weight, selection of SP resistance markers, and on ANC attendance and coverage of intermittent preventive treatment during pregnancy.

These project objectives will be achieved through multidisciplinary research comprising clinical and bio-medical research, health system research, socio-economic and anthropological studies with a focus on CHW, involving policy makers right from the start of the project as full project partners (http://www.cosmicmalaria.eu).

## Methods/designs

### Study areas and participant eligibility

This is a multi-centre trial involving three countries with varying malaria endemicity: low (The Gambia) versus high transmission (Burkina Faso and Benin), and varying degrees of SP resistance (high in Benin [19] and moderate in Gambia and Burkina Faso [20]). This will ensure that the study outcomes would be broadly generalisable in order to facilitate the formulation of recommendations for both the West African region and other sub-Saharan countries. The Gambia, a country where malaria transmission has decreased to levels thought not possible a few years ago (and hence in a so-called “epidemiological transition”), will provide information possibly applicable to countries that may reach a similar status in the (near) future.

In The Gambia, the study is situated in the eastern part of the country on the southern bank of the Upper River Region (about 170,000 inhabitants). It will be centred around the Basse Health Centre and satellite health facilities. This is an area of seasonal malaria transmission (July-December) that has recently experienced a significant decline in malaria infections. Nevertheless, malaria remains a significant problem with about 15% of pregnant women with placental malaria at delivery as detected by histopathology [9].

In Burkina Faso, the study is situated in the centre-west of the country in the Nanoro health district catchment area (about 145,000 inhabitants). Malaria transmission is high and extremely seasonal and overlaps with the rainy season (June-October).

In Benin, the study is situated at the Atlantic Region in the southern part of the country, in the Glo-Djigbé, Zinvié and Zè district communities (about 136,000 inhabitants). Malaria transmission is perennial and the main burden of the disease occurs during the two rainy seasons (April-July and October-November).

The current treatment policy of uncomplicated malaria in pregnancy is oral quinine but artemisinin-based combination therapy (artemether-lumefantrine (AL)) may be used in the second and third trimester of pregnancy for all three countries, or amodiaquine-artesunate in Burkina Faso and Benin [21–23].

All pregnant women resident in the study area and willing to remain so until delivery will be invited to participate. Women who have a history of sensitivity to sulphonamides and vulnerable persons (for example, the mentally deficient) will be excluded. In The Gambia, pregnant adolescents younger than 16 years will not be enrolled unless consent is given by a responsible adult. In Burkina Faso and Benin, a pregnant woman is considered an adult if married (regardless of age). Study participants are free to withdraw from the study at any time without giving a reason.

Prior to the trial start, community sensitization and involvement of village leaders will be carried out at each study site. Appropriate community meetings will be held where the trial will be explained to all community members and consent at the community level to participate from community leaders will be obtained. Individual signed informed consent will also be obtained from all participants.

### Design

**Figure 1:**
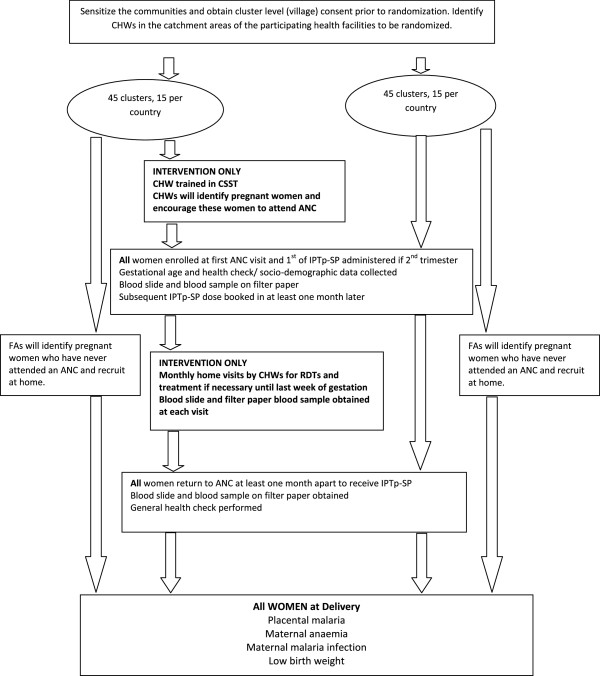
**Schematic of study design.** ANC, antenatal clinic; CHW, community health worker; CSST, community-scheduled screening and treatment; FA, field assistant; IPTp-SP, intermittent preventive treatment during pregnancy with sulfadoxine-pyrimethamine; RDT, rapid diagnostic test.

This is a multicenter, cluster-randomised controlled trial in which the CHWs and related catchment population (village/community) will be randomised to receive either the intervention (IPTp-SP plus CSST) or continue with current activities, that is IPTp-SP alone at health facilities during ANC visits (Figure 1).

### Study procedures

#### All communities

**Figure 2:**
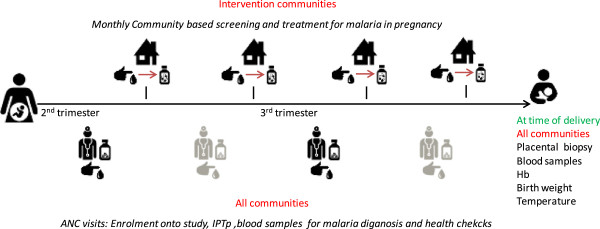
**Schedule of events.** Symbols are from thenounproject.com collection: ‘baby’ symbol (1), ‘baby’ symbol (2) by Edward Boatman, ‘house’ symbol by Marco Olgio, ‘medicine’ symbol by Emmanual Mangatia, ‘medicine’ symbol by Paulo Volkova, ‘doctor’ symbol by Paulo Volkova, ‘pointer’ symbol by Evan MacDonald and ‘drop’ symbol by Eric Bergholz. ANC, antenatal clinic; Hb, haemoglobin; IPTp; intermittent preventive treatment during pregnancy.

In principle, the first point of contact with pregnant women is at the first ANC visit. Here, the study will be explained to the woman and informed consent will be obtained. If the woman is in the early stage of pregnancy, she will be encouraged to return during her second trimester for IPTp-SP. If the woman is in the second or third trimester stage, health staff will give the first IPTp-SP dose and plan the second one onto the ANC card at least 1 month after the first dose. A data collection form on health and socio-demographic factors and a physical examination will be carried out, and a blood slide and a blood sample on filter paper will be collected for detection of malaria infection and SP resistance markers (Figures 1 and 2). All women will be encouraged to deliver at the health facility. If women do not attend the ANC, consent will be obtained at village level.

#### Intervention communities

CHWs allocated to the intervention arm will follow specific training on community-based case management of malaria, the need for monthly testing of pregnant women using a rapid diagnostic test (RDT), and on issues related to malaria in pregnancy, including the benefit of IPTp-SP and administering SP as early as possible in the second trimester of pregnancy.

The CHWs will continuously identify all pregnant women in their own catchment area, visit eligible women in their home and encourage them to attend the ANC as early as possible in their pregnancy for enrolment. The CHW will check after a week if the ANC was visited. For women who do not attend the ANC, the CHW will further encourage and discuss reasons for non-attendance.Thereafter, at monthly intervals up to the last week of gestation, the CHW will revisit the study participant’s house (Figure 2). The CHW will collect a blood slide and a blood spot on filter paper, perform a RDT and administer a full course of AL to any woman with a positive RDT. In case of treatment, the CHWs will return to assess uptake and compliance by administering a short questionnaire and checking the empty packaging of the treatment at the end of the course.

#### Control communities

In control communities, CHW will not be trained to administer RDT and AL to pregnant women. The women will be mainly followed through their attendance to the health facilities at the ANC.

#### At time of delivery for all communities

The final study visit will occur at the time of delivery. A blood sample will be collected just before delivery for haemoglobin measurement as well for detection of malaria infection and SP resistance markers and a short data collection form on their current health status will be administered. A placenta biopsy will be collected and all newborns will be physically examined and weighed as soon as possible after birth (Figures 1 and 2). Gestational age will be estimated using the Ballard Score [24]. For women not delivering at a health facility, study staff will follow them up at home and will collect the same information and biological samples as at the health facilities as soon as possible.

### Randomisation

The allocation of the two trial arms will be carried out separately but consistently for each country. Prior to the trial start, maps and population size of the study site in all three countries will be obtained. CHWs who work in villages with a population size of approximately 1,000 to 2,000 will be identified. If more clusters than required are identified, the appropriate number of representative clusters will be selected at random. Distance from the centre of each village to the nearest IPTp-SP-providing health facility will be calculated and used to group the clusters into three categories. Stratification by these categories will ensure that the villages with the larger distances are evenly distributed between trial arms. The balanced allocation of each cluster to a trial arm will be done using a computer-based randomisation (StataCorp LP, USA, http://www.stata.com).

### Clinical data collection and patient treatments

#### At the antenatal clinic

All study participants returning to the ANC for scheduled or unscheduled visits will undergo a health assessment by study staff. In case of suspected malaria (fever, headache, and so forth), a RDT test will be performed and, if positive, AL administered.

#### Community health worker home visits in the intervention arm only

RDT will be performed on a monthly basis for women enrolled in the intervention arm regardless of the presence of malaria symptoms. If positive for malaria, treatment will be given with AL. If the woman is severely ill at time of visit (from malaria or other causes and/or from complications from the pregnancy) she will be referred to the health facility for further care.

### Laboratory evaluations

#### Haematology

Maternal haemoglobin will be measured using Hb301 Hemocue*®* (part of the Radiometer Group, Sweden) and anaemia (haemoglobin <11 g/dL) diagnosed at the time of delivery.

#### Peripheral malaria infection

Blood samples will be collected by finger pricks at specified time points during the trial for blood slides (thick blood film) and blood spots on filter paper. Thick blood smears will be stained with 3% Giemsa for 30 minutes and read by trained microscopists at each site. Parasite densities will be calculated by counting the number of asexual parasites per 200 leukocytes (or per 500 leukocytes if the count is <10 asexual parasites/200 leukocytes), assuming a leukocyte count of 8,000/μl. A blood smear will be considered negative when the examination of 100 high-power fields does not reveal asexual parasites. Each slide will be read separately by two experienced microscopists and discrepancies resolved by a third reader.

Blood spots on filter paper will be used for the molecular diagnosis of *P. falciparum* (to detect sub-patent infections) and the identification of SP resistance markers.

#### Placental malaria

A 1 cm^2^ biopsy specimen will be obtained from the maternal-facing side of the placenta as soon as possible after delivery. Biopsy specimens will be preserved in 10% neutral buffered formalin which will be processed and embedded in paraffin wax by standard techniques. Pending histological evaluation, all biopsies will be kept at 4°C. Paraffin sections 4 millimeters thick will be stained with hematoxylin-eosin stain.

Placental biopsies will be classified according to the following definitions [25]:

1. Acute infection (parasites present, malaria pigment absent)
2. Chronic infection (parasites and malaria pigment present)
3. Past infection (no parasites but pigment present)
4. No infection (both parasites and malaria pigment absent)

### Safety considerations

The risk of participating in this cluster randomized trial is low. The World Health Organization recommends the use of artemisinin-based combination therapy, in this case AL, during the second and third trimester of pregnancy [26, 27]. The subjects will not be exposed to any danger or increased risk due to the conduct of the study. There is the possibility of mild discomfort and bruising at the site where blood is obtained. Collection of blood samples is part of the normal procedure in the diagnosis of malaria and is considered not to be a medical risk when adequately performed by a qualified health worker (including CHWs). Placental biopsies will be obtained after delivery, with no risk or discomfort for the neonate or mother. Qualified personnel using appropriate disposable equipment will collect blood samples and placental biopsies.

CHWs in the intervention arm will be trained on safety reporting requirements and on the recognition of danger signs and serious adverse events (SAEs). In case of any SAE of the pregnant woman, deterioration of her condition or reaction after the administration of the drug, the CHW will report this immediately to the study staff who will perform the necessary further assessments and referrals to the appropriate health facilities if required.

During the course of the study, any important medical events which occur will be appropriately recorded, managed and reported. Midwives and/or research staff will record all occurrences of adverse pregnancy outcomes such as a miscarriage or spontaneous abortion (<28 weeks of gestation), stillbirth (>28 weeks) or the occurrence of any congenital malformation. All reported SAEs and important medical events will be reported to the study site investigator or deputy. The local principal investigator will inform the sponsor and the coordinating investigator. The sponsor will inform the ethics committee and the Data Safety and Monitoring Board.

### Study endpoints

#### Primary endpoint

Prevalence of placental malaria (any category, including past infection) determined by a placental biopsy taken soon after delivery at a health facility.

#### Secondary endpoints

- Maternal anaemia (haemoglobin <11 g/dL) at delivery
- Maternal *P. falciparum* peripheral infection (microscopy and PCR) at delivery and during pregnancy
- Low birth weight (<2,500 grams)
- IPTp-SP coverage by village/cluster
- SP resistance markers profiles
- Number of ANC visits during pregnancy
- Number of doses of IPTp-SP taken during pregnancy

### Sample size rationale

The study will test the primary hypothesis that CSST plus IPTp-SP will reduce placenta malaria compared to IPTp-SP alone. Sample size calculation was performed according to Hayes and Bennett [28]. Based on available data, the prevalence of placental malaria (any category, including past infection) is at least 15% in the three study sites. Although the coefficient of variation for placental malaria is not known, it is set at 0.15 within each country. It is assumed that the intervention will decrease placental malaria by 30%, from 15% to 10.5%. Furthermore, the assumption is made that each CHW will recruit at least 40 pregnant women per year, corresponding roughly to a population of 800 people (assuming 5% of the population is pregnant). Within each study site, the study will be able to show a significant difference between intervention and control groups with 15 clusters per arm at 80% power and at the 10% significance level. This translates to 45 clusters per arm across the three study sites. Therefore, the total study population will be 5,400 women, 1,800 women in each country, and will require an 18-month recruitment period in order to enrol at least 60 women per cluster.

### Data handling and record keeping

All data handling and record keeping will be standardised across the three study sites. At the health facility, all pregnant women will be recorded on a study register held at each clinic. Women who are enrolled onto the study will also be given a unique identity number.

A standardised data collection form on the health status, medical history of the women, and socio-demographic characteristics will be administered by health staff at the time of enrolment at the health facility. A standardised data collection form on current health status will then be administered at subsequent ANC visits (intervention and control arm) and at each monthly visit by the CHWs in the intervention arm only. All data will be double entered using OpenClinica® (http://www.openclinica.com) databases at each site. Consistency checks will be performed and any outliers and missing data points will be checked against the original forms and subsequently amended in the dataset. Errors including missing data that may have occurred during the data collection will be reported with a minimum delay for further investigation.

All names and addresses will be removed from the data collection forms before data entry. The datasets will be password protected and only accessed by the data manager and local investigators at each study site. All samples will be labelled with the woman’s unique identity number and date of collection. This will ensure anonymity so all handlers of the specimens are blinded to the intervention allocation.

### Analytical plan

Appropriate summary statistics (that is, mean and standard deviation for normally distributed variables and median and inter-quartile range for non-normally distributed variables) will be used to describe the baseline data.

The primary analysis will compare the prevalence of placental malaria (defined as number of positive samples by total number of samples) between the intervention and the control group.

Secondary analysis will compare the following variables between trial arms: (i) mean haemoglobin levels and prevalence of anaemia (haemoglobin <11 g/dL) at delivery, (ii) prevalence of *P. falciparum* peripheral infection (microscopy and PCR) at delivery and during pregnancy, (iii) mean birth weight and proportion of newborns with low birth weight (<2,500 g) between the two trial arms, (iv) level of ANC attendance by village/cluster and coverage of IPTp-SP (defined by number of doses completed during pregnancy), and (v) SP resistance markers profiles.

For the primary and secondary analysis both linear regression (for continuous outcomes such as haemoglobin levels) and logistic regression (for binary outcomes such as placental malaria) will be used to quantify the difference between trial arms. The regression models will allow for the multi-level structure of the data (that is, individuals within villages) using random effects and for all relevant co-variates to be taken into account. Model assumptions will be checked to confirm reliable estimates.

### Timetable of event

The recruitment of study subjects will last for 18 months. The expected duration for each study subject is approximately 6 months - from enrolment during pregnancy up to time of delivery. It is expected that the total duration of the trial will be 24 months to allow the delivery of the last recruited pregnant woman.

### Ethical approval

Ethical approval by the Gambia Government/MRC laboratories Joint Ethics Committee was obtained on 25 June 2013 (ref SCC1336). Ethical approval by the Institutional Ethics Committee of Centre Muraz in Burkina Faso was obtained on 19 September 2013 (ref A20-2013/CE-CM). Ethical approval by the Comité National d’Ethique pour la Recherche en Santé in Benin was obtained on 9 December 2013 (N^o^_0126/MS /DC/SGM /DFR /CNERS/SA).

## Discussion

To reach successful global malaria control, there is an urgent need to access those at greatest risk of malaria infection. IPTp-SP if given at least twice after the first trimester has been shown to be effective in preventing malaria infection and its consequences during pregnancy. However, in the majority of sub-Saharan African countries, IPTp-SP coverage is low, with only 22% of pregnant women receiving IPTp-SP [16]. Systematic screening of pregnant women for malaria, and treating them if positive may further reduce the burden of malaria in pregnancy. In Ghana, an area of moderately high transmission, screening by RDT at scheduled ANC visits and treating positive women was as effective as IPTp-SP [29]. However, both interventions are currently being implemented through health facilities and thus the health system essentially waits for the women to attend a facility and does not tackle the issue of low or untimely (late in pregnancy) attendance. Further issues with delivery of these interventions include: unclear policy and guidance of IPTp-SP, stock outs at health facilities for both bed nets and SP, and poor quality of care. Low IPTp-SP coverage was also associated with poor education, poor knowledge of malaria, socio-economic status and age [30]. In rural areas, this is a major issue as pregnant women often have limited access to health facilities due to distance, poverty, low levels of education, strong traditional values, limited access to information and poor road infrastructure [31, 32]. Long distances can be an actual barrier for attending a health facility but they can also be a deterrent to seeking care [33].

In this study, it is proposed to add an “extension” strategy to the communities in rural areas to the standard IPTp-SP delivered through the health facilities and thus bring health services closer to where women live. The study will build on the use of CHWs who are already managing malaria cases within the framework of CCMm [18]. At this level, RDTs for the diagnosis of malaria are already being used or their implementation is planned. CCMm supported by RDT is at various stages of implementation; in The Gambia, the National Malaria Control Program is planning to introduce nationwide CCMm supported by RDT; in Burkina Faso, CCMm has been implemented and is currently managed by an international non-government organisation (Plan International) and four local non-government organisations under the supervision of the National Malaria Control Program; in Benin, CCMm is part of the National Malaria Policy, though it still needs to be scaled up nationwide.

The study will extend the CCMm which at present is primarily aimed at children under the age of 5 years to pregnant women. The aim is to improve both IPTp-SP coverage as CHWs encourage women to attend the ANC for the IPTp at the right time, allow systematic screening for malaria infection and provide an opportunity to treat malaria when the prophylactic effect of IPTp-SP has waned. The trial will also investigate the prevalence of SP resistance markers in the positive malaria samples obtained during the study as it could be hypothesized that the beneficial effects of the intervention might be more pronounced in areas with high SP resistance, because women could be more prone to placental malaria when IPTp-SP has a reduced effect and, although not yet observed in West Africa, extremely high levels of SP resistance markers in the parasite population may even lead to adverse effects of SP [34].

## Trial status

At time of submission of this paper, ethical approval has been obtained, and recruitment is ongoing in all three study sites.

## Abbreviations

AL: artemether-lumefantrine
ANC: antenatal clinic
CCMm: community case management of malaria
CHW: community health worker
CSST: community-scheduled screening and treatment
IPTp-SP: intermittent preventive treatment during pregnancy with sulfadoxine-pyrimethamine
PCR: polymerase chain reaction
RDT: rapid diagnostic test
SAE: serious adverse event.
